# Molecular and Sociodemographic Colorectal Cancer Disparities in Latinos Living in Puerto Rico

**DOI:** 10.3390/genes14040894

**Published:** 2023-04-11

**Authors:** Julyann Perez-Mayoral, Maria Gonzalez-Pons, Hilmaris Centeno-Girona, Ingrid M. Montes-Rodríguez, Marievelisse Soto-Salgado, Belisa Suárez, Natalia Rodríguez, Giancarlo Colón, Javier Sevilla, Daphne Jorge, Xavier Llor, Rosa M. Xicola, Doris H. Toro, Luis Tous-López, Marla Torres-Torres, José S. Reyes, Nicolas López-Acevedo, Ajay Goel, Segundo Rodríguez-Quilichini, Marcia Cruz-Correa

**Affiliations:** 1 NCI Center for Cancer Genomics, Bethesda, MD 20892, USA; 2 University of Puerto Rico Comprehensive Cancer Center, San Juan, PR 00936, USA; 3 School of Medicine, Universidad Central del Caribe, Bayamon, PR 00956, USA; 4 School of Medicine, Ponce Health Sciences University, Ponce, PR 00716, USA; 5 Department of Internal Medicine and Digestive Diseases, School of Medicine, Yale University, New Haven, CT 06520, USA; 6 VA Caribbean Healthcare System, San Juan, PR 00921, USA; 7 Ashford Presbyterian Community Hospital, San Juan, PR 00907, USA; 8 Beckman Research Institute, City of Hope Comprehensive Cancer Center, Duarte, CA 91010, USA; 9 Department of Medicine, Medical Sciences Campus, University of Puerto Rico, San Juan, PR 00935, USA

**Keywords:** colorectal cancer, Hispanics, early-onset colorectal cancer, molecular markers

## Abstract

Background: The incidence of sporadic colorectal cancer (CRC) among individuals <50 years (early-onset CRC) has been increasing in the United States (U.S.) and Puerto Rico. CRC is currently the leading cause of cancer death among Hispanic men and women living in Puerto Rico (PRH). The objective of this study was to characterize the molecular markers and clinicopathologic features of colorectal tumors from PRH to better understand the molecular pathways leading to CRC in this Hispanic subpopulation. Methods: Microsatellite instability (MSI), CpG island methylator phenotype (CIMP), and *KRAS* and *BRAF* mutation status were analyzed. Sociodemographic and clinicopathological characteristics were evaluated using Chi-squared and Fisher’s exact tests. Results: Of the 718 tumors analyzed, 34.2% (*n* = 245) were early-onset CRC, and 51.7% were males. Among the tumors with molecular data available (*n* = 192), 3.2% had MSI, 9.7% had *BRAF*, and 31.9% had *KRAS* mutations. The most common *KRAS* mutations observed were G12D (26.6%) and G13D (20.0%); G12C was present in 4.4% of tumors. A higher percentage of Amerindian admixture was significantly associated with early-onset CRC. Conclusions: The differences observed in the prevalence of the molecular markers among PRH tumors compared to other racial/ethnic groups suggest a distinct molecular carcinogenic pathway among Hispanics. Additional studies are warranted.

## 1. Introduction

Colorectal cancer (CRC) is the second leading cause of cancer death among men and women in the United States (U.S.) [1]. Disparities in CRC incidence and survival have been well documented among racial/ethnic groups in the mainland U.S. [2,3]. Although CRC incidence and mortality rates are lower among U.S. mainland Hispanics than in other racial/ethnic groups, aggregating heterogeneous populations (e.g., Hispanics) may mask the significant variability in CRC incidence and mortality within subgroups [4]. For example, among the Hispanic subgroups living on the U.S. mainland, Cubans and Puerto Ricans have disproportionately higher CRC incidence and mortality rates [5,6]. In Puerto Rico, CRC is the leading cause of cancer death in men and women [7].

During the past 30 years, the incidence of sporadic, non-familial CRC among individuals <50 years (early-onset CRC) has been increasing at an alarming rate in the U.S. and is expected to increase by >140% by 2030 [8,9,10]. In the U.S., more than 11% of CRC cases and 6% of deaths were reported due to early-onset CRC during 2012–2017 [11]. During the same period in Puerto Rico, more than 9% of the CRC cases and approximately 6% of CRC deaths corresponded to patients <50 years old [7]. Despite the attention the ascending CRC incidence rates among young individuals have garnered, the molecular events that lead to the development of early-onset CRC remain poorly understood.

Sporadic CRC is a heterogeneous disease that arises from the gradual accumulation of genetic and epigenetic alterations, some of which can be used as molecular markers to inform medical treatment decisions (i.e., MSI, *BRAF* or *KRAS* mutations) [12]. The CpG island methylator phenotype (CIMP) is another molecular feature associated with CRC [13]; however, the role of the CIMP in colorectal carcinogenesis is still not clearly understood. Based on the combination of these molecular markers (MSI, CIMP, and *BRAF* and *KRAS* mutations), colorectal tumors can be classified according to three carcinogenic pathways [14,15]. The traditional pathway, which leads to approximately 50–70% of all CRC cases, is characterized by chromosomal instability and APC and *KRAS* mutations resulting in CIMP-negative and MSI-low or microsatellite stable (MSS) tumors with a predominant distal location. Up to 30% of colorectal tumors are estimated to arise from the alternate carcinogenesis pathway, where *KRAS* or *APC* mutations precede the development of CIMP-low, MSI-low, or MSS tumors. In the serrated pathway, a *BRAF* mutation leads to MSI-high, MSI-low, or MSS, CIMP-high tumors that comprise 10–20% of all CRC cases [16].

There is limited information regarding the pathways that lead to CRC among Hispanics, as most studies have focused on non-Hispanic Whites and non-Hispanic Blacks [17,18]. This study describes the molecular and clinicopathological characteristics of CRC tumors in Hispanics living in Puerto Rico (PRH), a Hispanic subpopulation with a disproportionate CRC burden. A better understanding of the molecular events leading to colorectal carcinogenesis in Hispanic subpopulations, such as PRH, is necessary to develop tailored prevention and/or treatment strategies to promote health equity and reduce CRC mortality.

## 2. Materials and Methods

### 2.1. Patient Recruitment, Sociodemographic, and Clinicopathological Characteristics

A total of 718 sporadic, non-familial colorectal tumors from PRH were recruited through the Puerto Rico Familial Colorectal Cancer Registry. This island-wide, population-based registry recruits and collects biospecimens from individuals with gastrointestinal neoplasia and healthy controls. Fresh frozen tumor tissues were collected during tumor resection and stored at −150 °C for future analysis. According to the U.S. Census, “Hispanic or Latino” is defined as a person of Cuban, Mexican, Puerto Rican, South or Central American, or other Spanish culture or origin regardless of race. All participants in the Puerto Rico Familial Colorectal Cancer Registry complete a questionnaire (in Spanish) modeled from the one used in the Collaborative Family Registries for Colorectal Cancer. This questionnaire collects information on which country the participant was born in as well as the country where their father and mother were born, allowing us to classify subjects as Hispanic.

For this study, exclusion criteria included having a diagnosis of any hereditary genetic syndrome (i.e., familial adenomatous polyposis, Lynch syndrome) or inflammatory bowel disease. Only adenocarcinomas confirmed by pathology were included. The sociodemographic data analyzed included: age at recruitment (<50 vs. ≥50 years of age), gender (female vs. male), marital status (single/divorced/widowed vs. living together/married), educational level (<high school diploma or equivalent vs. ≥high school diploma or equivalent), type of health insurance (private vs. public vs. Medicare/Medicare Advantage), current drinker (yes vs. no) and current smoker (yes vs. no). The clinicopathological characteristics examined were body mass index (BMI kg/m^2^; <25, underweight/normal vs. ≥25, overweight/obese), family history of CRC (yes vs. no), tumor location (proximal vs. distal vs. unknown), tumor differentiation (high vs. moderate or low), and tumor stage (I/II vs. III/IV).

### 2.2. Genomic DNA Extraction

Genomic DNA colorectal tissue was extracted using a Gentra Puregene Tissue Kit (Qiagen, Germantown, MD, U.S.), following the manufacturer’s protocol. DNA concentrations were quantified using a Nanodrop (Thermo Scientific, Waltham, MA, U.S.). Samples with 260/280 ratio of ~1.8 were subsequently subjected to PCR analysis using *β-actin* primers to assay DNA integrity. Samples from which *β-actin* could not be amplified were excluded from the study. A representative agarose gel electrophoresis assay showing amplification of the expected 586 bp amplicon is shown in Supplementary Figure S1.

### 2.3. MSI, KRAS, and BRAF Status

Microsatellite instability (MSI) status was obtained by analyzing tumor samples and corresponding normal mucosa as previously described [19,20]. The MSI testing panel used included the following markers: BAT-25, BAT-26, BAT-40, D2S123, D5SS346, D175250, and TGFBR2. These markers included the reference panel of five MSI markers recommended by the National Cancer Institute for colorectal tumors; loci were scored according to published guidelines [21]. The mutation status of *KRAS* and *BRAF* was obtained from pathology reports or by performing a custom real-time PCR assay using a qBiomarker Somatic Mutation PCR Array (cat # 337021) following the manufacturer’s instructions (Qiagen, Germantown, MD, U.S.). This somatic mutation PCR array was a custom TaqMan-based mutation panel designed specifically to a restricted number of mutations in *KRAS* and *BRAF*, including: 1 *BRAF* (COSMIC ID: COSM476 or V600E (cat # SMPH001828A) and 11 *KRAS* somatic mutations (COSMIC ID: COSM516 (cat #SMPH007535A), COSM517 (cat #SMPH007533A), COSM520 (cat #SMPH007537A), COSM521 (cat #SMPH007531A), COSM522 (cat #SMPH007536A), COSM527 (cat #SMPH007541A), COSM531 (cat #SMPH007589A), COSM532 (cat #SMPH007538A), COSM553 (cat #SMPH007544A), COSM554 (cat #SMPH007540A), COSM555 (cat #SMPH007546A)). The master mix consisted of a 2X qBiomarker Probe Mix, genomic DNA (50 ng/μL), and RNase-free water, in a 25 μL total reaction volume. The real-time PCR was carried out in a StepOnePlus™ Real-Time PCR System (Applied Biosystems, Waltham, MA, U.S.) with the following cycling parameters: 95 °C for 10 min, 40 cycles of 95 °C for 15 s, 60 °C for 60 s. Supplementary Figure S2 shows a quantification plot of one sample for all the analyzed mutations.

### 2.4. CpG Island Methylation Phenotype (CIMP) Analysis

Bisulfite conversion was performed on 300 ng of genomic tumor DNA using a methylSEQr Kit (Applied Biosystems, Waltham, MA, U.S.) following the manufacturer’s protocol. A total of 2 μL of the bisulfite-modified DNA was used for the subsequent methylation-specific PCR (MSP) analysis performed using primers specific for the eight genes in the CIMP panel: *CAGNA1G, CRABP1, NEUROG1, IGF2, RUNX3, SOCS1, CDKN2 and MLH1* [22,23]. The conditions for the MSP were as follows: 95 °C for 10 min, followed by 40 cycles of 94 °C for 30 s, the annealing temperature of the primer set for 40 s, 72 °C for 40 s, with a final extension of 72 °C for 10 min. Annealing temperatures and primer sequences for each gene in the CIMP panel are described in Supplementary Table S1. After MSP, PCR products were visualized in 2% agarose gel and documented using the Gel Doc 1000 system with molecular analysis software (Bio-Rad, Hercules, CA, U.S.). Tumors were classified according to CIMP status as follows: CIMP-Zero (0 methylated genes), CIMP-Low (one to five methylated genes), and CIMP-High (six to eight methylated genes) [23].

### 2.5. Ancestry Informative Markers (AIMS) Panel Genotyping

PBL genomic DNA was used to genotype 105 AIMs panels using the Sequenom MassArray iPLEX platform (Sequenom, San Diego, CA, U.S.) as described in Perez-Mayoral et al. 2019. This AIMs panel consists of SNP markers that inform European, African, and Amerindian ancestry and has been validated for estimating continental ancestry information in admixed Latino populations, including Puerto Ricans. Sequenom TYPER software (Sequenom, San Diego, CA, U.S.) was used to make genotype SNP calls. STRUCTURE v2.3 software (Stanford, CA, U.S.) was used to calculate individual ancestry estimates for each participant using a model-based clustering method.

### 2.6. Integrated Pathways

Tumors were classified according to their molecular characteristics into the traditional pathway (MSS, CIMP-negative, and/or *BRAF* and *KRAS*-wild type), serrated pathway (*BRAF*-mutated, CIMP-positive, and any MSI or MSS), alternative pathway (MSS, CIMP-L, and *KRAS*-mutated), or other pathway [15,24,25]. Cases with unavailable data for the analyzed markers were classified into the traditional, serrated, or alternate pathways if two or more markers were available. Otherwise, cases were classified as other pathway.

### 2.7. Statistical Analysis

Frequency distributions and percentages were generated to describe the sociodemographic and clinicopathological characteristics of the sample. The differences between the variables were evaluated using Fisher’s exact test or the Chi-squared test. All statistical analyses were performed using STATA 16.0 (StataCorp LLC, College Station, TX, U.S.). Comparisons between PRH and other racial/ethnic groups were tested using a two-sample proportion test (prtesti). A *p*-value < 0.05 was used to determine statistical significance. This value indicates the presence of significant differences among the compared groups. However, given the cross-sectional nature of the study, inferences on causality are not possible.

## 3. Results

### 3.1. Description of the Study Population

A total of 718 subjects with sporadic CRC were recruited during 2007–2017. Among the cases included in the study, 34.2% of the subjects were diagnosed with CRC before 50 years of age (early-onset CRC), 51.7% were male, 60.7% had ≥12 years of education, and most had European admixture (Table 1). Most of the tumors evaluated were from CRC patients with no family history of CRC (72.3%), were in the distal colon (72.6%), and were diagnosed at early stages (stage I or II; 71.9%).

### 3.2. Description of the CRC Tumors with Molecular Markers

Of the 718 tumors, we had molecular marker data for 192 cases. Molecular testing of CRC tumors showed that *BRAF*- and *KRAS*-mutation status, MSS, and CIMP overall age distribution were similar among those diagnosed with early-onset CRC (< 50 years) and later-onset CRC (50 years) (Table 2). A higher frequency of wild-type *KRAS* was observed among later-onset CRC cases (72.9% *p* = 0.077); these results were marginally significant. A slightly higher frequency of MSI-high (5.6%) and CIMP-low (92.3%) was observed among early-onset CRC cases. MSI was only detected in 6 out of 186 cases, and 1 out of 111 cases had the CIMP-high phenotype.

### 3.3. BRAF/KRAS Mutation Status

The *KRAS* mutation spectrum was evaluated in 30 CRC tumors (Table 3). The most common somatic mutations found were G12D (26.6%) and G13D (20.0%). Both of these changes were predicted to be pathogenic with a pathogenicity score of 0.98 based on the Functional Analysis through Hidden Markov Models (v2.3) in silico model [26]. 

### 3.4. CRC Pathways

Of the 186 CRC tumors with two or more molecular markers, the majority (49.5%) were classified into the “other pathways” category (Table 4). When comparing the CRC pathways according to ancestry, a significantly higher number of cases had a higher percentage of Amerindian admixture in the traditional and other pathways (*p* = 0.029). A higher number of individuals with tumors from the alternate pathway reported < high school diploma or equivalent education (54.3%, *p* < 0.05).

### 3.5. Characteristics According to CRC Diagnostic Age

Considering the increasing trend in early-onset CRC incidence, we compared the sociodemographic and clinicopathological characteristics according to age at diagnosis (*n* = 717; Table 5). A higher percentage of females was diagnosed with early-onset CRC (58.4%) compared to later-onset CRC (43.2%; *p* < 0.05). A significantly lower rate of European admixture and higher Amerindian admixture was detected in individuals younger than 50 years diagnosed with CRC. Compared to individuals with later-onset CRC, those younger than 50 years of age when diagnosed with CRC had higher educational levels, private insurance, and no family history of CRC (*p* < 0.05).

### 3.6. Comparison of BRAF, KRAS, Microsatellite Instability and CIMP Status among Different Populations

Differences in the prevalence of the studied markers and the gender variable were found when comparing PRH with other global populations (Table 6). Compared to PRH, the Spanish and U.S. populations had a higher proportion of males by 7.3% and a lower proportion of females by 7.3% and 6.7%, respectively, across both genders. PRH had a higher prevalence of *BRAF* mutations (9.7%) than what was reported among the Spanish (6.2%; *p* < 0.05), Chinese (0.7%; *p* < 0.001), and two U.S. cohort studies, the Nurses’ Health Study (NHS) and the Health Professionals Follow-up Study (HPFS) (14.6%; *p* > 0.05) [27,28,29]. The prevalence of *KRAS* mutations in PRH (31.9%) was higher than in the Chinese group (24.9%) but lower than in the Spanish (36.9%) and U.S. cohorts (35.9%), although these differences were not statistically significant. CIMP-H status was markedly lower in PRH (0.9%) compared to the Spanish, Chinese, and U.S. populations (27.4%, 10%, and 17.5%, respectively). In addition, the MSI-H status of PRH was lower than that of the Chinese population (6.1%; *p* > 0.05) and significantly lower than that of the U.S. cohort (15.4%; *p* < 0.001) [27,28,29].

## 4. Discussion

Although CRC incidence and mortality rates are lower among U.S. mainland Hispanics than in other racial/ethnic groups, CRC continues to be one of the leading causes of cancer mortality among Hispanics [30]. Racial/ethnic early-onset CRC disparities have also been reported, with Hispanics having the most marked increases in early-onset CRC incidence annually [31,32]; however, the molecular events leading to the development of early-onset CRC and the racial/ethnic disparities observed remain poorly understood. One of the limitations in most studies that include Hispanics is that individuals from different countries of origin are usually classified together as “Hispanics”, which may mask the significant variability in CRC incidence and mortality within subgroups [4]. In this study, we report the molecular characterization of CRC tumors from Puerto Rican Hispanics, the second-largest Hispanic subpopulation in the mainland U.S., for the first time.

Analysis of the molecular biomarker data available for tumors in our PRH showed that the frequency of *BRAF* mutations, MSI, CIMP status, and *KRAS* mutations was different from what was reported for tumors from individuals from other racial/ethnic groups. The frequency of *BRAF* mutations found in PRH tumors overall (9.7%) is noticeably lower than that in non-Hispanic Blacks (56%) and non-Hispanic Whites (11.5–44%) [33,34,35,36]. Previous reports have also established an association between *BRAF* mutations and MSI status [35,37,38]; this association was not observed in our cohort. Colorectal tumors in the proximal colon were reported to be more likely to have *BRAF* V600E mutations and the CIMP-high phenotype than tumors in the distal colon [39,40]. The majority (72.6%) of the tumors included in the study were in the distal colon, which could in part explain the low rates of *BRAF* mutations and CIMP-high tumors. Overall, *KRAS* mutation frequencies in our PRH cohort (31.9%) are lower than those reported in other racial/ethnic groups, including non-Hispanic Blacks (59%) and non-Hispanic Whites (37–41%) [33,34,35,41]. A previous study in a separate cohort of 501 PRH reported a slightly higher but comparable prevalence of *KRAS* mutations (39%) [42]. The percentage of tumors with MSI in our cohort was markedly lower (4.3%) than what has been reported in African Americans (14–19%) and non-Hispanic Whites (9–13%) [33,37,43,44]. A very low number of tumors in our cohort had CIMP, with only one case having the CIMP-high phenotype. This result is markedly lower than the reported prevalence of CIMP-high among non-Hispanic Whites (13%), non-Hispanic Blacks (4.5%), and Hispanics (12.3%) [45]. We compared the prevalence of tumor markers from PRH with that reported for the nationwide and multicenter Spanish EPICOLON I and EPICOLON II projects, a Chinese population study, and the U.S. cohort studies, NHS and HPFS [27,28,29]. PRH has a higher prevalence of *BRAF* mutations than the Spanish and Chinese populations, but a lower prevalence than the U.S. study cohort. The prevalence of *KRAS* mutations ranges from 24.9% to 35.9% across populations, with the U.S. having the highest prevalence and the Chinese having the lowest. When compared, the Spanish cohort had the highest CIMP-H status, while the PRH cohort had the lowest. On the other hand, the U.S. had the highest percentage of MIS-H cases, while PRH had the lowest. Importantly, both MSI-L and MSI-H were reported as MSI by the Spanish group. The differences in the frequencies of the tumor biomarkers in our study population and other racial/ethnic groups in the mainland U.S. and other global populations can be attributed to various factors, including genetic (e.g., population-specific variations) and environmental exposures and diet, among others [46,47,48]. Although Hispanics share a common language and history, according to the country of origin, Hispanic subpopulations have different diets, exposures, and degrees of European, African, and Native American genetic admixture, which may explain the differences in colorectal tumor biomarkers in our population [49,50].

Approximately 90% of the somatic mutations found in KRAS in colorectal cancer tumors are in codons 12, 13, and 61 [51,52]. The most common mutations in KRAS observed in our cohort were G12D and G13D; these two mutations were found to be pathogenic according to the FATHMM in silico model [26]. G12V and G12C, some of the most commonly reported G12 KRAS mutations [53], represented a low percentage of the KRAS mutations detected in our population. KRAS mutation status is currently used as a prognostic factor for anti-EGFR therapies [54]. Recent studies suggest that individuals with mutations in codon 13 could derive benefits from anti-EGFR therapy [55,56], but individuals with somatic mutations in KRAS codon 12 have been reported to have worse overall survival than individuals with other KRAS somatic mutations [57]. Although the KRAS mutation rates in our PRH cohort were markedly lower than what has been reported in non-Hispanic Blacks, the higher frequency of KRAS mutations in codon 12 could be a contributing factor to the comparable and significantly higher relative risk of CRC death reported for PRH and non-Hispanic Blacks compared to non-Hispanic Whites [2].

The genetic and epigenetic alterations that lead to colorectal carcinogenesis can be grouped into three major pathways: traditional pathway (MSS, CIMP-negative, and/or wild type BRAF and KRAS), serrated pathway (MSI or MSS, CIMP-positive, BRAF mutation), and alternate pathway (MSS, CIMP-low, KRAS mutation). Most of the tumors in our PRH cohort were classified into the other pathway category, supporting the theory that distinct population-specific variation, genetic/epigenetic aberrations, and environmental factors may contribute to the carcinogenic process. A significantly higher number of individuals with tumors in the traditional and other pathway had a higher percentage of Amerindian admixture. Information on the prevalence of CRC molecular pathways according to race/ethnicity and genetic admixture is lacking, and warrants investigation. Our group previously reported that PRH with higher levels of African ancestry were three times more likely to develop colorectal tumors located in the rectum [58]. These findings show that genetic ancestry may have a role in the molecular development of CRC and further studies are needed to fully elucidate its contribution to colorectal carcinogenesis in diverse populations in order to develop tailored screening and treatment strategies to improve CRC outcomes.

Among our population, significant differences in gender, family history, and genetic admixture were observed when comparing early-onset versus later-onset CRC (>50 years at diagnosis). A higher number of women was diagnosed with early-onset CRC compared to men. This is in contrast to a large nationwide study reporting that men have 16% higher incidence rates of early-onset CRC [59]. As most early-onset CRC cases in our study were diagnosed at early stages, a possible reason that may in part explain this disparity is that women have been reported to seek more healthcare compared to men [60]. However, environmental exposures, in utero exposures, or epigenomic factors, among others may contribute to the observed gender-specific difference among this early onset cohort. The significantly higher number of early-onset CRC cases with family history of CRC compared to later-onset cases observed in our population is consistent with previous studies showing that family history of CRC is associated with early-onset CRC [61] and that some common CRC risk variants are more strongly associated with early-onset CRC than later-onset CRC [62]. Significantly lower levels of European admixture and higher levels of Amerindian admixture were observed among individuals with early-onset CRC. Hispanics vary in their percentages of admixture of ancestral population and in the fact that they are the racial/ethnic subgroup in the U.S. with the highest increase in annual incidence rates from 2013–2018 [59]. Thus, larger studies evaluating the association between genetic admixture and early-onset CRC are needed to determine if this is a factor that could be used to identify individuals at higher risk of developing CRC at an early age and to develop tailored screening guidelines according to admixture.

The strength of this study is that it characterizes the sociodemographic and clinical characteristics, as well as the molecular markers, from CRC cases diagnosed from PRH, a Hispanic subpopulation with a high CRC cancer burden. All subjects were recruited through PURIFICAR, a population-based registry that receives direct referrals from physicians and surgeons across the island. However, this could create a selection bias, and the data presented may not be representative of the PRH population. Another limitation is that not all the CRC cases recruited had all the molecular biomarkers examined in this study performed as part of their clinical workup. We were unable to perform molecular testing on tumors if subjects were recruited into the registry after surgery and we did not have access to the tumor tissue. Molecular testing was performed on all the tumor tissue we had available at the time of the study. The size of various subgroups, such as CRC tumors with MSI-high, limited the statistical analysis performed for this study, thus warranting a study with a larger sample size to be able to perform robust subgroup analysis, as well as to evaluate lifestyle CRC risk factors among PRH. In addition, future studies including a more comprehensive array of ”omic” data are warranted for CRC tumors among PRH in order to classify tumors into the CRC consensus molecular subtypes [25] and for the development of personalized treatment strategies to improve outcomes.

To the best of our knowledge, this is the first study to characterize tumors from PRH patients using MSI, CIMP, and *KRAS* and *BRAF* mutation status. The observation that the prevalence of these molecular markers is markedly different from what has been reported in other racial/ethnic groups suggests distinct pathways for CRC carcinogenesis in Hispanic populations. Moreover, the lower percentage of European admixture and higher Amerindian admixture detected in PRH with early-onset CRC supports the need for additional studies with larger sample sizes to examine ancestry, genetics, epigenetics, and lifestyle to fully understand and characterize the factors contributing to the development of early- and later-onset CRC in Hispanic subpopulations.

## Figures and Tables

**Table 1:** Sociodemographic and clinicopathological characteristics of the study population.

Characteristics	n	%
**Age at CRC diagnosis (*n* = 717)**		
<50 years	245	34.2
≥50 years	472	65.8
**Gender (*n* = 718)**		
Male	371	51.7
Female	347	48.3
**Ancestry (*n* = 413)**		
African admixture	0.21 ± 0.01	
European admixture	0.61 ± 0.01	
Amerindian admixture	0.18 ± 0.003	
**Educational Level (*n* = 455)**		
<High school diploma or equivalent	179	39.3
≥High school diploma or equivalent	276	60.7
**Marital Status (*n* = 377)**		
Single/Divorced/Widowed	110	29.2
Living Together/Married	267	70.8
**Health Insurance (*n* = 482)**		
Private	173	35.9
Public	157	32.6
Medicare/Medicare Advantage	152	31.5
**Family History of CRC (*n* = 650)**		
Yes	180	27.7
No	470	72.3
**Current Drinker (*n* = 663)**		
No	497	75.0
Yes	166	25.0
**Current Smoker (*n* = 663)**		
No	623	94.0
Yes	40	6.0
**BMI (*n* = 704)**		
<25 (Underweight/Normal)	222	31.5
≥25 (Overweight/Obese)	482	68.5
**Location of CRC tumor (*n* = 533)**		
Proximal	119	22.3
Distal	387	72.6
Colon, unspecified	27	5.1
**Tumor Stage (*n* = 708)**		
I/II	509	71.9
III/IV	199	28.1
**Tumor Differentiation (*n* = 421)**		
High	115	27.3
Low/Moderate	306	72.7

Counts vary between the variables due to missing information.

**Table 2:** Frequency of tumor biomarkers among PRH according to age at CRC diagnosis.

Markers	<50 Years n (%)	≥50 Years n (%)	*p*-Value
***BRAF*** **(*n* = 134)**			
**Wild type**	39 (90.7)	82 (67.8)	0.999
**Mutation**	4 (9.3)	9 (9.9)	
***KRAS*** **(*n* = 144)**			
**Wild type**	28 (58.3)	70 (72.9)	0.077
**Mutation**	20 (41.7)	26 (27.1)	
**Microsatellite status (*n* = 192)**			
**MSS**	67 (93.1)	117 (97.5)	0.312
**MSI-Low**	1 (1.4)	1 (0.8)	
**MSI-High**	4 (5.6)	2 (1.7)	
**CIMP status (*n* = 111)**			
**None**	2 (7.7)	8 (9.4)	0.999
**Low**	24 (92.3)	76 (89.4)	
**High**	0 (0.0)	1 (1.2)	

Counts vary between variables due to missing information.

**Table 3:** Frequency of *KRAS* mutations among tumors from PRH.

COSMIC ID	Codon Affected	HGVS cDNA	HGVS Protein	No. of Times Observed
COSM516	12	c.34G > T	p.G12C	2
COSM517	12	c.34G > A	p.G12S	0
COSM520	12	c.35G > T	p.G12V	4
COSM521	12	c.35G > A	p.G12D	12
COSM522	12	c.35G > C	p.G12A	4
COSM527	13	c.37G > T	p.G13C	3
COSM531	13	c.38-39GC > AT	p.G13D	5
COSM532	13	c.38G > A	p.G13D	9
COSM553	61	c.182A > T	p.Q61L	2
COSM554	61	c.183A > C	p.Q61H	3
COSM555	61	c.183A > T	p.Q61H	1

**Table 4:** Sociodemographic and clinicopathological characteristics according to CRC pathway in PRH.

Characteristics	Pathways (*n* = 186)				
	Traditional *n* (%)	Serrated *n* (%)	Alternate *n* (%)	Other Pathway *n* (%)	*p*-Value
	*n* = 38	*n* = 14	*n* = 42	*n* = 92	
**Age at diagnosis** **(*n* = 186)**					
<50 years	16 (42.1)	5 (35.7)	17 (40.5)	38 (41.3)	0.986
≥50 years	22 (57.9)	9 (64.3)	25 (59.5)	54 (58.7)	
**Gender** **(*n* = 186)**					
Male	24 (63.2)	4 (28.6)	21 (50.0)	41 (44.6)	0.108
Female	14 (36.8)	10 (71.4)	21 (50.0)	51 (55.4)	
**Ancestry** **(*n* = 123)**					
African Admixture	0.20 ± 0.09	0.19 ± 0.05	0.24 ± 0.16	0.17 ± 0.07	0.307
European Admixture	0.62 ± 0.10	0.66 ± 0.05	0.61 ± 0.16	0.63 ± 0.10	0.582
Amerindian Admixture	0.18 ± 0.07	0.14 ± 0.06	0.15 ± 0.06	0.19 ± 0.07	0.029
**Educational Level** **(*n* = 144)**					
<High school diploma or equivalent	6 (19.4)	4 (36.4)	19 (54.3)	18 (26.9)	0.013
≥High school diploma or equivalent	25 (80.7)	7 (63.6)	16 (45.7)	49 (73.1)	
**Marital Status** **(*n* = 107)**					
Single/Divorced/Widowed	6 (30.0)	6 (54.6)	11 (32.4)	16 (38.1)	0.549
Living Together/Married	14 (70.0)	5 (45.5)	23 (67.7)	26 (61.9)	
**Health Insurance** **(*n* = 145)**					
Private	19 (57.8)	2 (22.2)	13 (34.2)	22 (33.9)	0.054
Public	5 (15.2)	5 (55.6)	9 (23.7)	25 (38.5)	
Medicare/Medicare Advantage	9 (27.3)	2 (22.2)	16 (42.1)	18 (27.7)	
**Family History of CRC (*n* = 179)**					
Yes	28 (75.7)	12 (92.3)	31 (77.5)	60 (67.4)	0.239
No	9 (24.3)	1 (4.7)	9 (22.5)	29 (32.6)	
**Current Drinker** **(*n* = 171)**					
No	25 (67.6)	12 (92.1)	29 (72.5)	54 (66.7)	0.294
Yes	12 (32.4)	1 (7.7)	11 (27.5)	27 (33.3)	
**Smoker** **(*n* = 171)**					
No	35 (92.1)	11 (84.6)	37 (94.9)	75 (92.6)	0.654
Yes	3 (7.9)	2 (15.4)	2 (5.1)	6 (7.4)	
**BMI** **(*n* = 185)**					
<25 (Underweight/Normal)	9 (23.7)	3 (23.1)	19 (45.2)	27 (29.4)	0.160
≥25 (Overweight/Obese)	29 (76.3)	10 (76.9)	23 (54.8)	65 (70.7)	
**Tumor location** **(*n* = 152)**					
Proximal	10 (27.8)	7 (50.0)	11 (29.0)	17 (26.6)	0.247
Distal	25 (69.4)	7 (50.0)	23 (60.5)	46 (71.9)	
Colon, unspecified	1 (2.8)	0 (0.0)	4 (10.5)	1 (1.6)	
**Tumor Stage** **(*n* = 184)**					
I/II	33 (86.8)	13 (92.9)	36 (85.7)	76 (84.4)	0.940
III/IV	5 (13.2)	1 (7.1)	6 (14.3)	14 (15.6)	
**Tumor Differentiation (*n* = 135)**					
High	11 (31.4)	2 (16.7)	10 (30.3)	11 (20.0)	0.511
Low/Moderate	24 (68.6)	10 (83.3)	23 (69.7)	44 (80.0)	

Counts vary between variables due to missing information.

**Table 5:** Sociodemographic and clinicopathological characteristics evaluated according to age at CRC diagnosis.

Characteristic	<50 Years	≥50 Years	*p*-Value
**Gender (*n* = 717)**			
Male	102 (41.6)	268 (56.8)	<0.001
Female	143 (58.4)	204 (43.2)	
**Ancestry (*n* = 412)**			
African Admixture	0.22 ± 0.10	0.20 ± 0.13	0.056
European Admixture	0.59 ± 0.12	0.62 ± 0.11	0.009
Amerindian Admixture	0.19 ± 0.07	0.17 ± 0.08	0.009
**Educational Level (*n* = 455)**			
<High school diploma or equivalent	42 (25.8)	137 (46.9)	<0.001
≥High school diploma or equivalent	121 (74.2)	155 (53.1)	
**Marital Status (*n* = 377)**			
Single/Divorced/Widowed	37 (27.6)	73 (30.0)	0.679
Living Together/Married	97 (72.4)	170 (70.0)	
**Health Insurance (*n* = 482)**			
Private	89 (53.0)	84 (26.8)	<0.001
Public	72 (42.9)	85 (27.1)	
Medicare/Medicare Advantage	7 (4.2)	145 (46.2)	
**Family History of CRC (*n* = 650)**			
Yes	76 (33.3)	104 (24.6)	0.018
No	152 (66.7)	318 (75.4)	
**Current Drinker (*n* = 662)**			
No	169 (74.1)	328 (75.6)	0.681
Yes	59 (25.9)	106 (24.4)	
**Current Smoker (*n* = 662)**			
No	214 (94.3)	408 (93.8)	0.806
Yes	13 (5.7)	27 (6.21)	
**BMI (*n* = 703)**			
<25 (Underweight/Normal)	76 (31.3)	146 (31.7)	0.900
≥25 (Overweight/Obese)	167 (68.7)	314 (68.3)	
**Tumor location (*n* = 532)**			
Proximal	40 (21.9)	79 (22.6)	0.835
Distal	135 (73.8)	251 (71.9)	
Colon, unspecified	8 (4.4)	19 (5.4)	
**Tumor Stage (*n* = 707)**			
I/II	169 (70.1)	339 (72.8)	0.462
III/IV	72 (29.9)	127 (27.3)	
**Tumor Differentiation (*n* = 420)**			
High	39 (27.5)	75 (27.0)	0.916
Low/Moderate	103 (72.5)	203 (73.0)	

Counts vary between variables due to missing information.

**Table 6:** Comparison of gender, *BRAF* and *KRAS* mutation status, microsatellite instability and CIMP status among different populations.

	Study Population						
Characteristic	PRH	Spanish	*p*-Value	Chinese	*p*-Value	US	*p*-Value
**Gender**							
Male	51.7% (717)	59.0% (*n* = 876)	0.004	57.0% (*n* = 84)	0.358	45.0% (*n* = 1253)	0.004
Female	48.3 % (*n* = 717)	41.0% (*n* = 876)		43.0% (*n* = 84)		55.0% (*n* = 1253)	
**Markers**							
*BRAF* mutated	9.7% (*n* = 134)	6.2% (*n* = 878)	0.130	0.7% (*n* = 401)	<0.001	14.5% (*n* = 1253)	0.128
*KRAS* mutated	31.9% (*n* = 144)	36.9% (*n* = 878)	0.247	24.9% (*n* = 401)	0.104	35.9% (1249)	0.342
**MSI Status**							
High	3.1% (*n* = 192)	9.1% (*n* = 878)	-	6.1% (*n* = 82)	0.246	15.4% (*n* = 1253)	<0.001
Low	1.0% (*n* = 192)		-	23.2% (*n* = 82)	<0.001		
MSS	98.9% (*n* = 192)	-	-	70.7% (*n* = 82)	<0.001	84.6% (*n* = 1253)	<0.001
**CIMP Status**							
None	9.0% (*n* = 111)	-	-	12.5% (*n* = 401)	0.311	43.6% (*n* = 1173)	<0.001
Low	90.0% (*n* = 111)	-	-	77.3% (*n* = 401)	0.003	38.9% (*n* = 1173)	<0.001
High	0.9% (*n* = 111)	27.4% (*n* = 878)	<0.001	10.2% (*n* = 401)	0.002	17.5% (*n* = 1173)	<0.001

## Data Availability

Data are available through a controlled access repository at https://crcweb.rcm.upr.edu/redcap/. Researchers are required to submit a web-based application to request access to the data. Once the request is approved, access will be granted.

## Supplementary Materials

The following supporting information can be downloaded at: https://www.mdpi.com/article/10.3390/genes14040894/s1, Table S1: Primer Information for CIMP Panel Genes, Figure S1: DNA integrity assessed by β-actin PCR analysis; Figure S2: TaqMan-based mutation panel analysis.
