# Supplementary material for: Molecular and Sociodemographic Colorectal Cancer Disparities in Latinos Living in Puerto Rico
Source: Genes (Basel). 2023 Apr 11;14(4):894. doi: 10.3390/genes14040894 (PMC10138302; doi:10.3390/genes14040894)

**Table S1: Primer Information for CIMP Panel Genes**

| Primer Pair | Forward | Reverse | AT |
|---|---|---|---|
| *CAGNA1G* UM | TTG GAG TTT GGG TGT GAA GTG A | CAC AAA TCC CAC TTC CCC TAC A | 55° |
| *CAGNA1G* M | GGA GTC GGT CGG TTG GTT C | AAA ACA TAC TAC CCG CGA AAC G | 60° |
| *IGF2* UM | GGA TTG TGG GTG TTT AGT TTG GTT | CCT TTC CAC ACT ACA TCC CAA AA | 58° |
| *IGF2* M | AGC GGT TTC GGT GTC GTT ATC | CGA ACG CCC AAC TCG ATT | 62° |
| *NEUROG1* UM | TTG TTG GTT AAT TGG TGG TGT TGT | CAT ACC TCA ACC ACT AAT CAC CCA | 55° |
| *NEUROG1* M | AAT TTA TGT TCG CGG GAG GTC | ACC AAC TTA ACC CGA ACC GA | 55° |
| *RUNX3* UM | TTT GGG TTT ATG GGA ATA TG | TTC TCA CAA CAA CAA CAA CC | 55° |
| *RUNX3* M | TGT TTT CGT TTA TTT TGT CG | CGC TAT TAT ACG TAT TCC CG | 55° |
| *SOCS1* UM | GAT GGT TGG AGT TAG AAT TGG TTG TT | CTC TAT ACT CCA CAA AAC TCT TCC CA | 60° |
| *SOCS1* M | GTA TTT TTT TGG TGC GCG ATA GTC | CGA CCG ACC TAA AAA TAC ACG C | 66° |
| *CDKN2A (p16)* UM | TTA TTA GAG GGT GGG GTG GAT TGT | CAA CCC CAA ACC ACA ACC ATA A | 56° |
| *CDKN2A (p16)* M | TAT TAG AGG GTG GGG CGG AT | ACC CCG AAC CGC GAC CGT AA | 58° |
| *CRAPB1* UM | GAG GTT TTT TAG TTG GAG AGT GG | AAC TCA CAA AAC AAA AAC TAA CAC T | 62° |
| *CRAPB1* M | GGA GGT TTT TTA GTT GGA GAG C | CTC GCA AAA CGA AAA CTA ACG | 63° |
| *MLH1* UM | TTT TGA TGT AGA TGT TTT ATT AGG GTT GT | ACC ACC TCA TCA TAA CTA CCC ACA | 60° |
| *MLH1* M | TAT ATC GTT CGT AGT ATT CGT GT | TCC GAC CCG AAT AAA CCC AA | 60° |

**Figure S1: DNA integrity assessed by β-actin PCR analysis.** This representative figure shows the electrophoretic profile of 5 DNA samples assayed for the amplification of the 586 bp β-actin fragment.

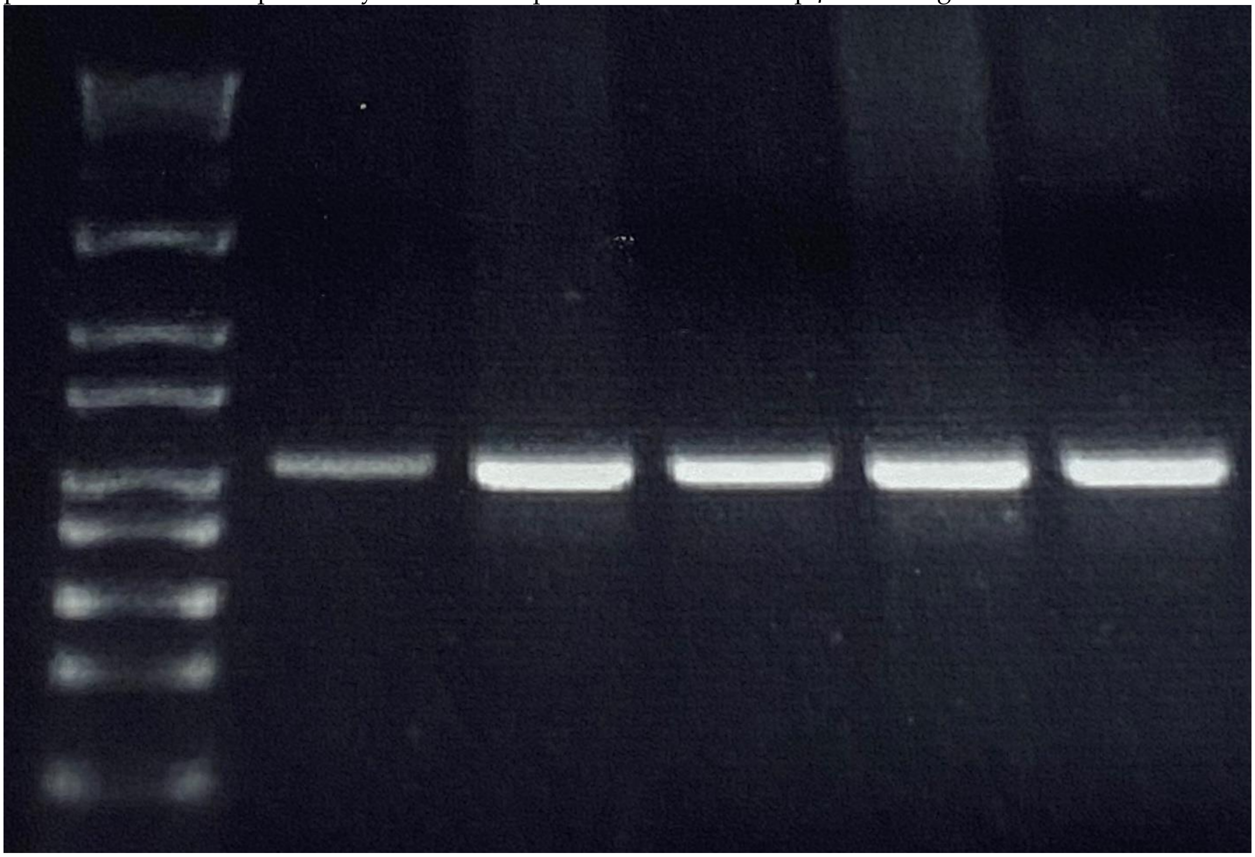

**Figure S2: TaqMan-based mutation panel analysis.** This representative amplification plot shows the mutation profile of a sample that was positive for the KRAS COSM553.

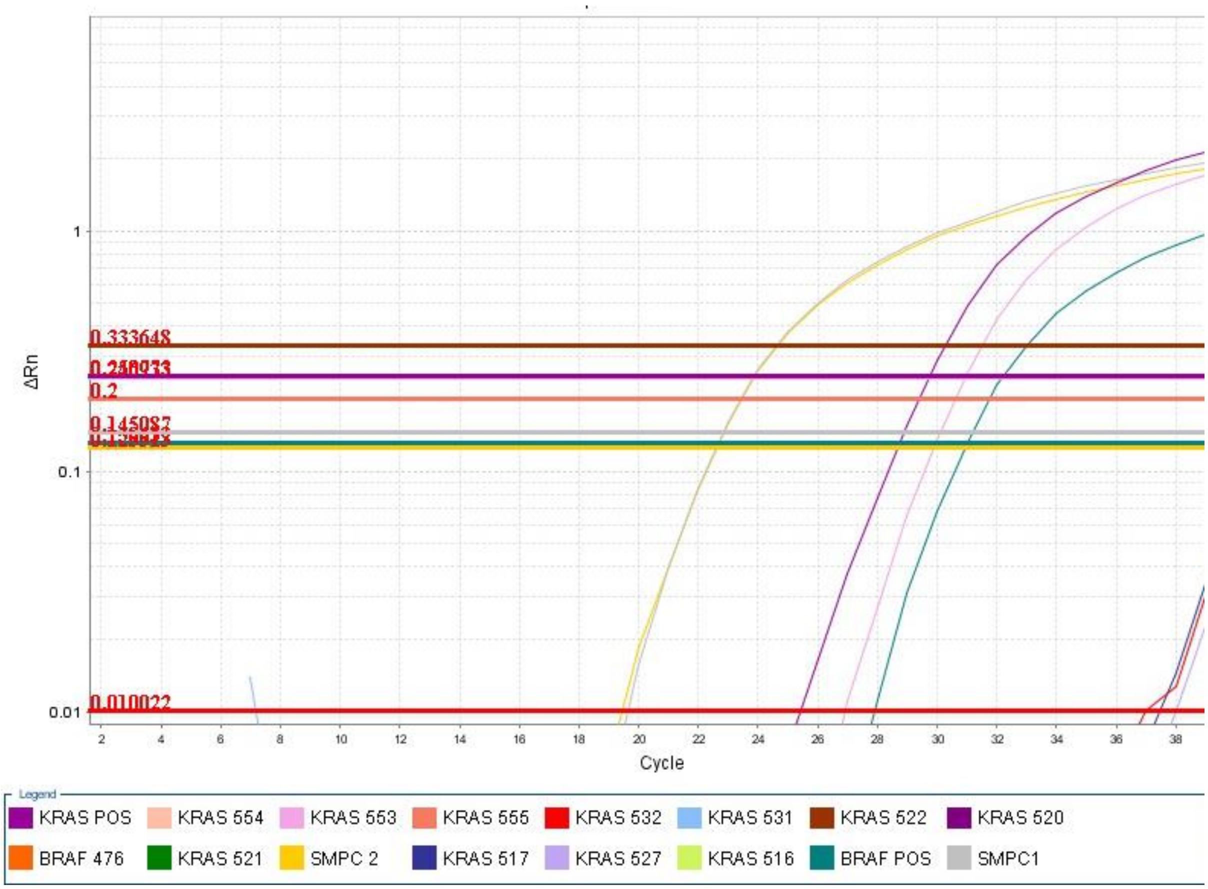

Supplement: Supplementary file 1 [file genes-14-00894-s001.zip › genes-2254812-supplementary.pdf]
